# Pathogenesis of IgA Vasculitis: An Up-To-Date Review

**DOI:** 10.3389/fimmu.2021.771619

**Published:** 2021-11-09

**Authors:** Yan Song, Xiaohan Huang, Guizhen Yu, Jianjun Qiao, Jun Cheng, Jianyong Wu, Jianghua Chen

**Affiliations:** ^1^ Kidney Disease Center, The First Affiliated Hospital, College of Medicine, Zhejiang University, Hangzhou, China; ^2^ Key Laboratory of Kidney Disease Prevention and Control Technology, Hangzhou, China; ^3^ National Key Clinical Department of Kidney Diseases, Hangzhou, China; ^4^ Institute of Nephrology, Zhejiang University, Hangzhou, China; ^5^ The Third Grade Laboratory Under the National State, Administration of Traditional Chinese Medicine, Hangzhou, China; ^6^ Department of Dermatology, The First Affiliated Hospital, College of Medicine, Zhejiang University, Hangzhou, China

**Keywords:** IgA vasculitis, IgA, pathogenesis, kidney, skin

## Abstract

Immunoglobin A (IgA) vasculitis (IgAV), formerly called the Henoch-Schönlein purpura (HSP), is a small vessel vasculitis, characterized by IgA1-dominant immune deposition at diseased vessel walls. IgAV is the most common form of vasculitis in children; typical symptoms include palpable purpura, arthritis or arthralgia, abdominal pain, and hematuria or proteinuria. Galactose-deficient IgA1 is detected in the tissues of the kidney and skin in patients with IgAV; it forms immune complexes leading to subsequent immune reactions and injuries. This report provides the recent advances in the understanding of environmental factors, genetics, abnormal innate and acquired immunity, and the role of galactose-deficient IgA1 immunocomplexes in the pathogenesis of IgAV.

## 1 Introduction

Immunoglobin A (IgA) vasculitis (IgAV), formerly called the Henoch-Schönlein purpura (HSP), is a small vessel vasculitis, characterized by IgA1-dominant immune deposition at diseased vessel walls (1). It may occur as systemic or single-organ limited vasculitis. The skin, kidney, gastrointestinal tract, and joints are often involved (1).

IgAV is the most common form of vasculitis in children, with an annual incidence rate of ~20 per 100,000 children (2–7). Typical symptoms include palpable purpura, arthritis or arthralgia, abdominal pain, and hematuria or proteinuria. In most cases, the disease is self-limited, but relapse is common. Gastrointestinal involvement occurs in 10%–40% of patients, and renal involvement occurs in 10%–55% of patients. Altogether, they are the principal causes of morbidity and mortality (8–10). IgAV is relatively rare with an incidence of 0.8–2.2 per 100,000 person-years in adults (2, 11). Aging negatively impacts the severity and outcome of the disease in adult patients with IgAV (12). Younger patients are more frequently involved with the joint and gastrointestinal tract, whereas old patients are at increased risk of severe purpura and glomerulonephritis, including end-stage kidney disease (ESKD) (12–16).

Although the epidemiology, clinical manifestations, and outcomes of IgAV are well established, our understanding of the pathogenesis of IgAV is still limited. In the past decade, efforts have been made to further understand IgAV. Identification of environmental and genetic factors and recognition of aberrant IgA may shed light on the pathogenesis of IgAV. Herein, we present recent advances in the understanding of the environmental factors, genetic factors, abnormal innate and acquired immunity, and the role of galactose-deficient IgA1 immunocomplexes in the pathogenesis of IgAV.

## 2 Environmental Factors and IgAV

The seasonal tendency of IgAV has been reported in several extensive cohort studies (17–19). The onset is more frequent during September to April and less common during summer. Recently, according to a Croatian study, geospatial clustering of IgAV along the course of Drava and Danube rivers, similar to the spatial distribution of Balkan endemic nephropathy triggered by daily exposure to environmental factors was observed (aristolochic acid) (20, 21). The temporal and geospatial associations imply that environmental factors are involved in the pathogenesis of IgAV.

The history of infection of the upper respiratory tract or the history of exposure to antigens from certain foods, insects, drugs, or vaccines can usually be found before the onset of IgAV, suggesting that infection or exposure to mucosal antigen may trigger the pathogenesis of IgAV (1, 22). This may also explain the regional and seasonal distribution of IgAV (23). The most commonly reported pathogens related to IgAV are group A *Streptococcus*, parainfluenza virus, and Human Parvovirus B19 (8, 17, 18). *Helicobacter pylori* is also associated with the disease. Patients with *H. pylori* have increased the risks of IgAV; *H. pylori* eradication therapy contributed to the rapid improvement of IgAV (24, 25). Since the COVID-19 outbreak, several cases of COVID-19 related IgAV have also been reported (26–28). The serum anti-COVID-19 IgA but not IgG was detected in patients with IgAV, and endothelial injuries may be involved (27). Besides pathogens, various vaccines, including the live attenuated vaccines of measles, mumps, rubella, and the inactive antigen vaccines of influenza or hepatitis B, may trigger IgAV (29).

The mechanism of pathogen or mucosal antigen-related IgAV is unclear; however, theoretically, it can be pointed to the modulation of mucosal immunity, including galactose-deficient IgA1 (Gd-IgA1) production (30–33). It was postulated that pathogen and mucosal antigens may trigger immune responses through molecular mimicry, increased intestinal permeability, and abnormal production of IgA1 results in subsequentially immune dysfunction (see details in the following section) (34, 35).

## 3 Genetics and IgAV

According to epidemiological studies across the world, incidence rates differ among races. In a UK study, Asians showed the highest incidence at 24.0(18.2–31.2) per 100,000 per year, whereas black people possessed the lowest incidence at 6.2 per 100,000 per year. Likewise, in a smaller population American study, the Hispanic children possessed a higher incidence (8.6 per 10,000 children) than African American and Caucasian children (0.9 per 10,000) (2, 3). In both studies, black people had a lower incidence than other ethnicities. However, epidemiological investigations were conducted *via* different methods, and further multicountry studies with uniform methodologies are needed to confirm whether ethnic variation is the risk factor of IgAV.

The genetic factors are related to IgAV (36). Human leukocyte antigen (HLA)-B35 and HLA-DRB1*01 alleles are associated with susceptibility to IgAV (37, 38). Especially, HLA-DR1*0103 is strongly associated with increased susceptibility to IgAV (39). Polymorphism in genes encoding cytokines was found to be associated with manifestations of IgAV (36). Polymorphism of IL-8, a cytokine that plays a central role in the recruitment of neutrophils, associates with an increased risk of cutaneous IgAV (40). IL-1 polymorphism was found to be associated with the severity and outcome of IgAV-N (41, 42). Polymorphism in enzyme encoding genes may also affect the synthesis of Gd-IgA1 (43, 44). Mutation of MTHFR gene (encodes methylenetetrahydrofolate reductase), and factor V Leiden may be associated with clinical symptoms (36, 45) (46). Association of Mediterranean fever (MEFV) gene mutation and IgAV has been reported in Mediterranean population (47–53). IgA vasculitis can occur at 2.7-7% patients with familial Mediterranean fever (46). These patients tends to have less IgA deposits than those with IgAV alone (54). MEFV gene encodes pyrin, a modulator of innate immunity, and pathogenic MEFV mutation leads to altered innate immune system inflammation and thus increases the susceptibility of vasculitis (55). The genetic susceptibility of IgAV may vary among different ethnic populations, and further investigation conducted across the world is warranted to confirm the role of these genes in different populations.

## 4 Immunopathogenesis of IgAV

As the name of the disease indicates, the most notable pathological feature of IgAV is IgA1-dominant IgA deposits in the vessel walls. Aberrant IgA and IgA complexes are considered to play a central role in the immunopathogenesis of IgAV.

IgAV shares many similarities with another IgA mediated disease, IgA nephropathy. (IgAN). IgAN is defined by the IgA-dominant deposits in mesangial area of the kidney. IgAV and IgAN shares many similarities in clinical and pathologic features. Especially, it can hardly be distinguished from renal-limited IgAV (56, 57). Similarities and differences between IgAV with nephritis and IgAN are presented in **Table 1** (58, 59).

**Table 1:** Differences and similarities between IgAV with nephritis and IgAN(48, 49).

Characteristics	IgA *Vasculitis* with nephritis	IgA Nephropathy
**Onset**	Children younger than 10 years of age	More common in adulthood
**Organs involved**	Systemic or single-organ limited (skin, kidney, joint, gastrointestinal tract, etc. )	Kidney
**Disease course**	Acute, with spontaneous resolution	Chronic and progressive
**Gender preference**	More common in male (about 2:1)	
**Abnormal IgA**	Galactose-deficient IgA1	
**Light microscopy**	Mesangial proliferation, endocapillary hypercellularity, segmental sclerosis, crescents	
**Immunofluorescence microscopy**	IgA1 dominant deposits in the glomerular mesangium	
**Outcome**	More severe in adults	

**Figure 1:**
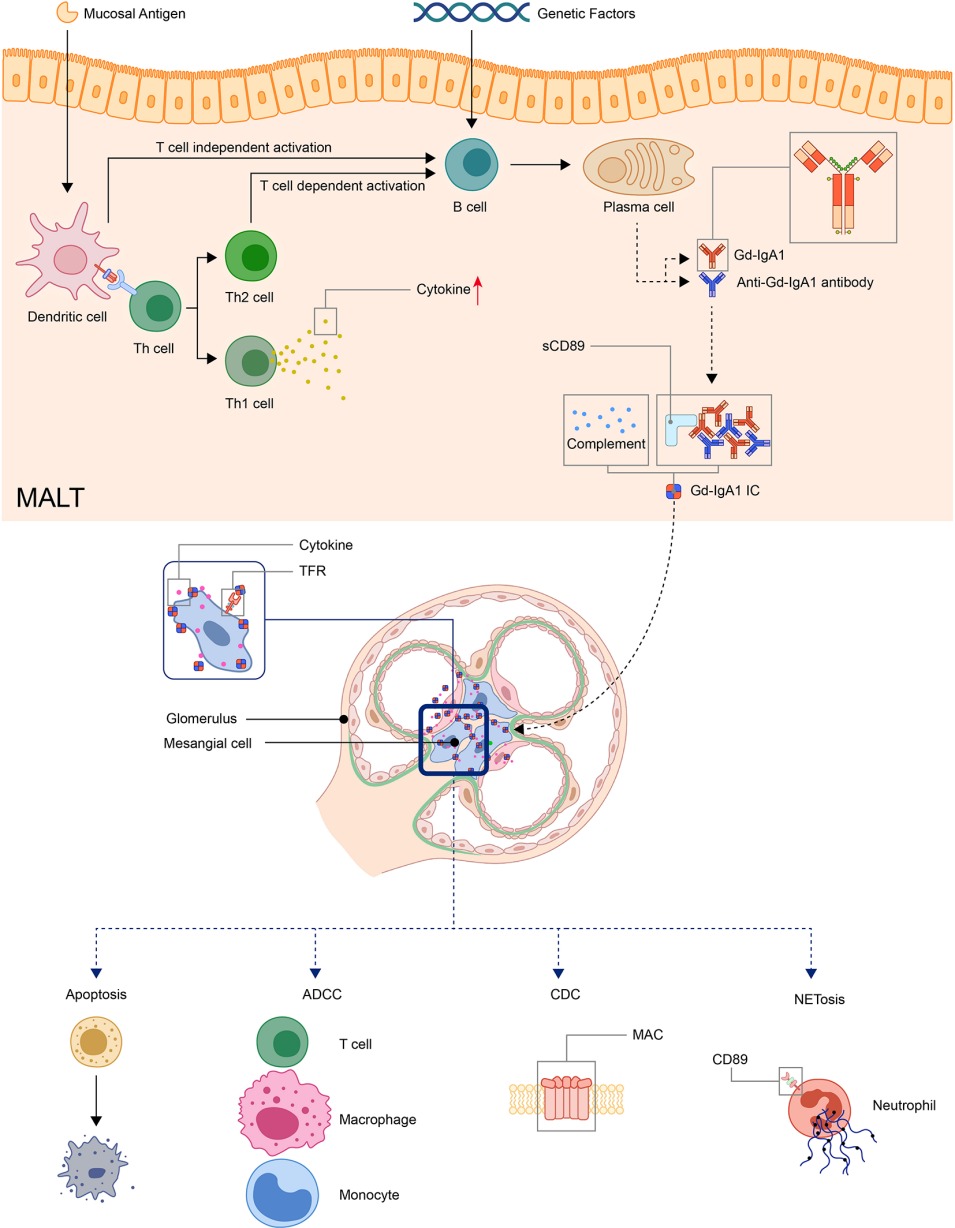
Model: pathogenesis of IgA vasculitis. The mucosal antigen can activate B-cells in MALT through T-cell-dependent or independent ways. The latter activates B-cells through TLR pathways. With genetic factors, the activated B-cells become plasma cells and produce Gd-IgA1. Gd-IgA1 and anti-Gd-IgA1 autoantibodies form circulating immune complexes together with other components (including sCD89 or complements). Then, the immunocomplex deposit at organs and activate inflammatory responses. In the kidney, the immunocomplex can activate mesangial cells through TfR, leading to the apoptosis of renal cells and recruitment of inflammatory cells. (ADCC, antibody-dependent cytotoxicity; CDC, complement-dependent cytotoxicity; Gd-IgA1, Galactose-deficient IgA1; MAC, membrane attack complex; MALT, Mucosa-associated lymphoid tissue; NET, neutrophil extracellular traps; TfR, transferrin receptor).

Detection of clustered onset in twins, one with IgAN and the other with IgAV-N, further confirmed the relationship between two diseases (60). So it has long been speculated that IgAV and IgAN may have similar pathogenic mechanisms (17). A widely accepted hypothesis for the pathogenesis for IgAN is a multi-hit model proposed by Novak J. et al (61). The model was originally used in IgA nephropathy but later found applicable in IgAV (62, 63). In this model, the first and second hit is the production of Gd-IgA1 and autoantibodies against Gd-IgA1, the third hit is the formation of Gd-IgA1 containing immune complexes, and finally, the fourth hit is the deposition of immune complexes in tissue activates the inflammatory process that results in organ injury (62–65) (**Figure 1**).

### 4.1 Galactose-Deficient IgA1 and Its Autoantibodies

The hallmark histologic feature of IgAV is leukocytoclastic vasculitis with IgA immune complex deposits in small vessels. Alterations in the O-linked glycosylation of IgA1 are found in patients with IgAV, and higher serum Gd-IgA1 level is related to a higher risk of kidney involvement, although not with disease severity (63, 66, 67). Gd-IgA1-dominant IgA deposits were detected in the kidney, skin, and gastrointestinal tract biopsies, and it was considered an important factor in the immunopathogenesis of IgAV (1, 68).

**Figure 2:**
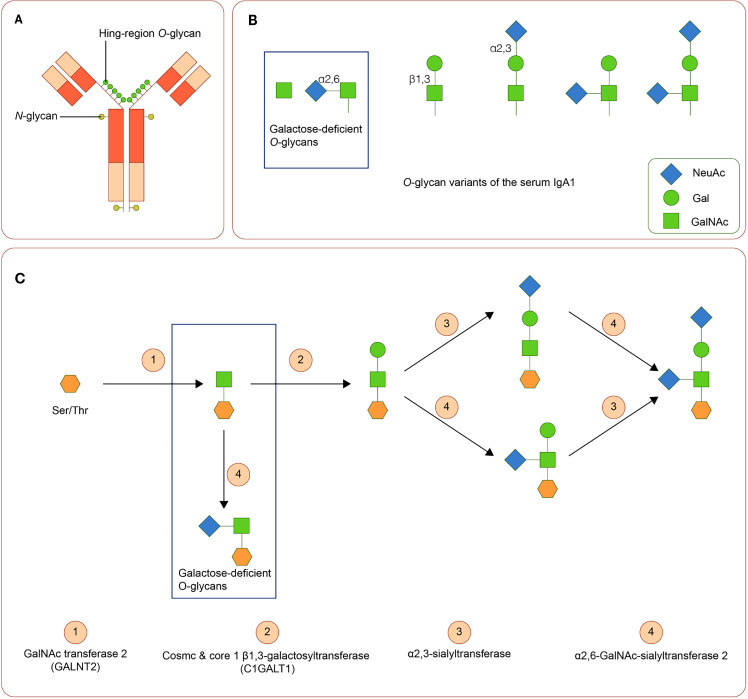
IgA1 Oglycans **(A)** Structure of human IgA1. The hinge region of IgA1 usually contains three to six O-linked glycan sites. **(B)** Variants of IgA1 O-glycan. **(C)** Synthesis of human IgA1 O-glycans. Upregulated α-N-acetylgalactosaminide α2,6-sialyltransferase 2 and down regulated core 1 β1,3-galactosyltransferase (and Cosmc) can lead to the increase of galactose-deficient IgA1.

IgA is a Y-shaped immunoglobulin with two heavy chains and two light chains. A short segment of amino acids forms the hinge region in the central part of the heavy chains. In general, according to its location, IgA can be subdivided into the serum or the secretory IgA. Serum IgA1 is predominantly produced by B-cells in the bone marrow, whereas secretory IgA is primarily generated by activated B-cells near the mucosae and the exocrine glands (69). Two subclasses of IgA, namely, IgA1 and IgA2, are produced in a ratio of 5:1. The hinge region of IgA1 usually contains three to six O-linked glycan sites (**Figures 2A, B**). At these sites, galactose (Gal) and N-acetylgalactosamine (GalNAc) with or without sialic acid (N-acetylneuraminic acid, Neu5ac) can attach to oxygen atoms of serine or threonine residues through glycosidic linkages (70, 71)

Variation in activities or expression of critical enzymes that catalyze O-glycosylation of IgA1 in B-cells may lead to Gal deficiency of IgA1. Three enzymes are critical in O-glycosylation of IgA1: polypeptide *N*-acetylgalactosaminyltransferase 2 (GALNT2) attaches GalNAc to serine or threonine, core 1 β1,3-galactosyltransferase (C1GALT1), with its chaperone Cosmc links Gal to GalNAc, and sialyltransferases complete the glycan structure by attaching sialic acid to Gal or GalNAc residues (α2,3-sialyltransferase for Gal and α2,6-GalNAc-sialyltransferase 2 for GalAc) (72). Downregulated expression of C1GALT1 and upregulated α2,6-GalNAc-sialyltransferase 2 are associated with the production of Gd-IgA1 (73). (**Figure 2C**). Cytokines, including IL-6 and IL-8, participate in the regulation of these enzymes (74). Polymorphisms in the genes of these enzymes are also involved in the synthesis of Gd-IgA1 (43, 44, 75–77).

As stated above, the onset of both IgAV and IgAN typically follows episodes of respiratory infection, which indicates that mucosal antigens may play a role in the process. The mucosal immune response can induce Gd-IgA1 production by peripheral B-cells. In mucosa-associated lymphoid tissue (MALT), activated B-cells produce IgA in T-cell-dependent or -independent manners. The latter one involves the interaction between B-cell and dendritic cell and the Toll-like receptor (TLR) pathway. Tonsillar B-cell activation through TLR on dendric cells may lead to the production of Gd-IgA1 in patients with IgAN, and tonsillectomy can reduce the serum levels of Gd-IgA1 (78, 79). TLR9 and the A proliferation-inducing ligand (APRL), IL-6 mediated pathways, and the TLR7-GALNT2 axis are involved in the synthesis of Gd-IgA1 (79, 80). TLR2 and TLR4 are upregulated in patients with IgAV and IgAN, and the level of TLR4 expression is related to proteinuria (81, 82).

The presentation of autoantibodies can be induced by the residues in Gd-IgA1 or mucosal antigen that mimic the structure of Gd-IgA (83). In Gd-IgA, the abnormal glycosylation exposes nearby GalNAc residues, and the latter can become neoepitopes (84). An elevated level of IgG autoantibodies specifically against Gd-IgA1 was detected in IgAV-N patients and is associated with disease activity, whereas in those without nephritis, it is similar to the control groups (85). There are other isotypes of autoantibodies against Gd-IgA1, but their role is poorly understood.

Reduced galactosylation in the O-glycan site of the hinge region of IgA1 was detected in patients with IgAV, especially IgAV-N (63, 86, 87). Recent findings of Gd-IgA1in the cutaneous lesions and clinically uninvolved skin in skin-limited IgAV further confirmed its role in IgAV (62). The serum level of Gd-IgA1–specific IgG is associated with disease activity and renal involvement (85). Targeted release formulation of budesonide that can target Payer’s patches in the ileum and suppresses the gastrointestinal immune system can decrease the level of Gd-IgA1 and may be a potential treatment for IgAV (88–90).

### 4.2 Formation of Immune Complex

The formation of the Gd-IgA1 immune complex is a critical step in the pathogenesis of IgAV. The proliferation of mesangial cells can be stimulated by Gd-IgA1 immune complexes, but not isolated Gd-IgA1 (91). The levels of serum Gd-IgA1 are heritable, and healthy relatives of patients may have elevated serum Gd-IgA1 levels without clinical symptoms, suggesting that Gd-IgA1alone is insufficient to cause IgAV; other factors, such as the formation of Gd-IgA1 immune complexes, are also critical for the pathogenesis of IgAV (92, 93).

Gd-IgA1 can self-aggregate or bind to its autoantibodies, thereby forming circulating immune complexes (CIC). IgA1 containing CIC is detected in patients with IgAV, although heterogeneity in CIC composition was observed (87). Patients without nephritis tend to have IgA1-CIC with smaller molecular mass, whereas that of those with IgAV-N is larger (usually IgA1-IgG-CIC) (85, 94, 95).

Fc alpha receptor I (FcαRI, also known as CD89) is a transmembrane IgA receptor in myeloid cells and can be released as a soluble form (sCD89) after cleavage of the extracellular domain. CD89 is involved in the deposition of IgA-CICs in the kidney. The serum levels of the sCD89-IgA1 complex are higher in IgAV patients than those in health control (96, 97). Transgenic mouse model expressing human CD89 and IgA1 suggested that IgA1 and sCD89 forms CICs that deposit at mesangial cells. sCD89 has a low affinity for monomeric or dimer IgA but a high affinity for IgA immune complexes (98). It can bind to IgA1 and further increase the size of IgA1-CIC. The interaction between CD89 and IgA-containing immune complexes results in phagocytosis, antibody-dependent cellular cytotoxicity, complement-dependent cytotoxicity, production of reactive oxygen species, and cytokines that lead to the destruction of the tissue (98–101).

### 4.3 Deposition of Immune Complex

The formation of IgA-CIC hinders the liver clearance of these immune complexes, and overloaded IgA-CIC can deposit at vessel walls (102–105). Deposition of Gd-IgA1 containing immunocomplex can be found in small vessel walls in the skin as well as the kidney and mesangial cells (62). The serum level of Gd-IgA1 is not correlated with the intensity of Gd-IgA1 deposits in the kidney and skin, suggesting that factors other than size and amount of CIC influence the deposition of immune complexes and other mechanisms are involved in the deposition of Gd-IgA1 (65).

#### 4.3.1 Deposition of Immune Complex in the Kidney

Collaboration between IgA-sCD89, transferrin receptors(TfR), and transglutaminase 2 is required in renal injury (106). TfR, also known as CD71, are a group of IgA1 receptors expressed in mesangial cells. In IgAV-N, TfR expression is increased. IgA1 isolated from patients with IgAV induces increased expression of TfR, activates PI3K/Akt/mTOR pathway, and stimulates the proliferation of human mesangial cells (107). Hypogalactocylation in IgA1 and large molecular sizes enhance the affinity of immune complexes to TfR in mesangial cells and promotes the deposition in mesangial cells and subsequent activation of the IgA receptor (102). β-1,4-galactosyltransferase, as an IgA receptor in mesangial cells, also participates in the deposition of IgA (108).

In patients with IgAV, immune complexes activate mesangial cells and induce mesangial proliferation, expression of proinflammatory cytokines and chemokines (IL-6, IL-8, TNFs, and MCP-1), and apoptosis of podocytes and tubular epithelial cells. These results in the recruitment of inflammatory cells, and further augmenting the injury in the kidney (61, 71, 109, 110).

#### 4.3.2 Deposition of Immune Complex in the Skin

It is controversial whether Gd-IgA1 participates in the pathogenesis of IgAV patients without nephritis. Traditionally, it is believed that IgG-containing CICs were found only in IgAV patients with nephritis, which tends to have poorer outcomes. And some clinical studies suggested that the serum levels of Gd-IgA1 or Gd-IgA1-CIC in IgAV patients without nephritis are the same against healthy controls (63, 86). However, in a recent study, Gd-IgA1 was detected using KM55 staining in the skin of IgAV patients without nephritis (62). The result suggested that Gd-IgA1 are important in both systemic and organ limited IgAV. Nevertheless, KM55 staining is a lectin-independent approach used to detect Gd-IgA1 (111). Gd-IgA1 deposits were also found using the staining method in renal biopsies in other secondary IgA nephropathy and incidental IgA deposits without nephritic syndrome (112, 113). The conclusion needs to be carefully explained and to be confirmed using different staining methods.

### 4.4 The Role of Complements

The activation of complements is also involved in tissue injuries in patients with IgAV. Elevated C3a, C5a, and Bb fragments, and C3 and C5-9 deposits, indicate the activation of the alternative pathway (114). C4d and C5b-9 deposits in the kidney are associated with poor renal outcomes (115).

Activation of complements through the mannose-binding lectin pathway was reported in IgA-V (116–118). A recent case report suggests that a monoclonal antibody against mannose-binding lectin serine peptidase 2, an inhibitor of the lectin pathway, can be used to treat IgAV-N (119). The activated complements induce upregulated expression of cytokines and recruit inflammatory cells (83, 93).

### 4.5 Inflammatory Cells in IgAV

Together with IgA immune complexes, infiltration of inflammatory cells can be observed around vessel walls, suggesting that these cells may be involved in tissue injury of IgAV.

#### 4.5.1 Neutrophils and NETs

Neutrophils are predominant cells in inflammatory infiltration in cutaneous and gastrointestinal biopsies from patients with IgAV. In neutrophils, the cross-link induces the release of neutrophil extracellular traps (NETs) and neutrophil chemoattractant leukotriene B4 that may augment the damage in a positive feedback manner (120, 121). NETs are web-like chromatin structures that play an important role in the clearance of pathogen. NETs are released through NETosis, and the latter is triggered by immune receptors through mediators, including reactive oxygen species (122). The immune complex can induce NETosis *via* FcγRIIIB or CD89 (123, 124). NETs and reactive oxygen species are increased in both superficial and deep dermal perivascular tissue in IgAV (125).

#### 4.5.2 T-Cells

T-cells are involved in tissue damage in IgAV (126, 127). There are two major populations of T-cells, CD4 and CD8 T-cells. CD4 T-helper (Th), CD4 regulatory T-cells (Treg), and CD8 cytotoxic T-lymphocytes (CTL) are important subsets of T-cells. Activation of circulating CTLs is found in patients with IgAV, and increased CTLs in glomeruli contribute to kidney injuries in IgAV (126). CXCR3 is highly expressed in effector T-cells. CXCR3-expressing T-cells are found recruited in the skin and kidneys of patients with IgAV, and the degree of infiltration of T-cell in the kidney is associated with the severity of kidney impairment (127).

Th17 is a subset of CD4-positive T-cells that produces IL-17. In patients with active IgAV, serum levels of IL-17 were elevated, and the number of Th17 in peripheral blood increased (128, 129). Interestingly, a monoclonal antibody against IL-17A (secukinumab) can trigger IgAV (130). It is speculated that the secukinumab breaks the balance of regulators of Th17 cells, leading to increased proinflammatory cytokines that induce IgAV. A recently reported case of IgA vasculitis complicated by psoriasis vulgaris may help explore the mechanism. In this patient, skin lesions of IgAV appears in sparing area of psoriasis (131). The patient adopted maxacalcitol, which can induce regulatory T-cells, and resident regulatory T-cells in psoriatic lesions may suppress the activity of IgAV.

Treg is a subset of T-cells that can suppress or regulate immune responses. Type-1 T regulatory (Tr1) cells can produce high levels of IL-10 and TGF-β, and are thought to regulate local immune microenvironments wherein specific antigens exist (132, 133). In patients with IgAV, the suppressive function of Tr1 is impaired, and the number of Tr1 in peripheral blood during remission is negatively associated with the relapse of the disease (134).

#### 4.5.3 Others

TNF-α is a cytokine mainly secreted by monocyte/macrophages. Serum TNF-α is elevated in patients with IgAV, and the level is associated with disease severity, suggesting the participation of other inflammatory cells (135). Paradoxically, TNF-α inhibitors may induce IgAV in patients with inflammatory bowel disease or psoriasis, but the causality is not confirmed (136–138). A possible explanation is that TNF-α inhibitors form immune complexes with endogenic TNF-α and deposits at vessel walls.

## Conclusion and Perspectives

Genetic factors, disrupted mucosal immunity, and immune complexes with abnormal IgA or IgA antibodies are essential in the pathogenesis of IgAV. Nevertheless, the pathogenic mechanisms of IgAV are far from being completely understood, and further investigations are required. For example, mucosal immunity, especially gastrointestinal lymphoid organs, may play a key role in the pathogenesis of IgAV. However, the cellular and molecular mechanisms are unclear; how pathogen or antigen triggers immune responses and what roles those cytokines recognized as biomarkers play in the pathogenesis is not known. Furthermore, the role of IgA in the development of vasculitis needs to be further explored. An in-depth understanding of how acquired and innate immunity participates in the pathogenesis of IgAV may provide the possibility of targeted treatments. Deciphering the molecular pathogenesis of IgAV can provide a platform to identify new targets for the treatment of the disease.

## Author Contributions

JHC, JW, and JC were responsible for the conception and design of the review. YS and XH drafted the manuscript. GY and JQ revised the manuscript. All authors contributed to the article and approved the submitted version.

## Funding

This work was supported by the Zhejiang Medical and Health Science and Technology Project (2020KY117).

## Conflict of Interest

The authors declare that the research was conducted in the absence of any commercial or financial relationships that could be construed as a potential conflict of interest.

## Publisher’s Note

All claims expressed in this article are solely those of the authors and do not necessarily represent those of their affiliated organizations, or those of the publisher, the editors and the reviewers. Any product that may be evaluated in this article, or claim that may be made by its manufacturer, is not guaranteed or endorsed by the publisher.

## References

[1] Jennette JC Falk RJ Bacon PA Basu N Cid MC Ferrario F. 2012 Revised International Chapel Hill Consensus Conference Nomenclature of Vasculitides. Arthritis Rheum (2013) 65(1):1–11. doi: 10.1002/art.37715 23045170

[2] Piram M Mahr A. Epidemiology of Immunoglobulin A Vasculitis (Henoch-Schonlein): Current State of Knowledge. Curr Opin Rheumatol (2013) 25(2):171–8. doi: 10.1097/BOR.0b013e32835d8e2a 23318735

[3] Gardner-Medwin JMM Dolezalova P Cummins C Southwood TR. Incidence of Henoch-Schonlein Purpura, Kawasaki Disease, and Rare Vasculitides in Children of Different Ethnic Origins. Lancet (2002) 360(9341):1197–202. doi: 10.1016/s0140-6736(02)11279-7 12401245

[4] Dolezalova P Telekesova P Nemcova D Hoza J. Incidence of Vasculitis in Children in the Czech Republic: 2-Year Prospective Epidemiology Survey. J Rheumatol (2004) 31(11):2295–9. 15517648

[5] Piram M Maldini C Biscardi S De Suremain N Orzechowski C Georget E. Incidence of IgA Vasculitis in Children Estimated by Four-Source Capture-Recapture Analysis: A Population-Based Study. Rheumatol (Oxford) (2017) 56(8):1358–66. doi: 10.1093/rheumatology/kex158 28444335

[6] Pillebout E Sunderkotter C. IgA Vasculitis. Semin Immunopathol (2021) 43(5):729–38. doi: 10.1007/s00281-021-00874-9 34170395

[7] Mossberg M Segelmark M Kahn R Englund M Mohammad AJ. Epidemiology of Primary Systemic Vasculitis in Children: A Population-Based Study From Southern Sweden. Scand J Rheumatol (2018) 47(4):295–302. doi: 10.1080/03009742.2017.1412497 29409373

[8] Hetland LE Susrud KS Lindahl KH Bygum A. Henoch-Schonlein Purpura: A Literature Review. Acta Derm Venereol (2017) 97(10):1160–6. doi: 10.2340/00015555-2733 28654132

[9] Gendreau S Porcher R Thoreau B Paule R Maurier F Goulenok T. Characteristics and Risk Factors for Poor Outcome in Patients With Systemic Vasculitis Involving the Gastrointestinal Tract. Semin Arthritis Rheum (2021) 51(2):436–41. doi: 10.1016/j.semarthrit.2021.03.002 33711774

[10] Nossent J Raymond W Keen HI Preen D Inderjeeth C. Morbidity and Mortality in Adult-Onset IgA Vasculitis: A Long-Term Population-Based Cohort Study. Rheumatol (Oxford) (2021). doi: 10.1093/rheumatology/keab312 33779729

[11] Tracy A Subramanian A Adderley NJ Cockwell P Ferro C Ball S. Cardiovascular, Thromboembolic and Renal Outcomes in IgA Vasculitis (Henoch-Schonlein Purpura): A Retrospective Cohort Study Using Routinely Collected Primary Care Data. Ann Rheum Dis (2019) 78(2):261–9. doi: 10.1136/annrheumdis-2018-214142 30487151

[12] Audemard-Verger A Pillebout E Baldolli A Gouellec NL Augusto JF Jourde-Chiche N. Impact of Aging on Phenotype and Prognosis in IgA Vasculitis. Rheumatol (Oxford) (2021) 60(9):4245–51. doi: 10.1093/rheumatology/keaa921 33410479

[13] Clave S Sordet M Tsimaratos M Decramer S Fila M Guigonis V. Association of Kidney Biopsy Findings With Short- and Medium-Term Outcomes in Children With Moderate-to-Severe IgA Vasculitis Nephritis. Eur J Pediatr (2021) 180(10):3209–18. doi: 10.1007/s00431-021-04065-4 33934234

[14] Audemard-Verger A Terrier B Dechartres A Chanal J Amoura Z Le Gouellec N. Characteristics and Management of IgA Vasculitis (Henoch-Schönlein) in Adults: Data From 260 Patients Included in a French Multicenter Retrospective Survey. Arthritis Rheumatol (2017) 69(9):1862–70. doi: 10.1002/art.40178 28605168

[15] Huang X Ma L Ren P Wang H Chen L Han H. Updated Oxford Classification and the International Study of Kidney Disease in Children Classification: Application in Predicting Outcome of Henoch-Schonlein Purpura Nephritis. Diagn Pathol (2019) 14(1):40. doi: 10.1186/s13000-019-0818-0 31077245 PMC6511170

[16] Van de Perre E Jones RB Jayne DRW. IgA Vasculitis (Henoch-Schonlein Purpura): Refractory and Relapsing Disease Course in the Adult Population. Clin Kidney J (2021) 14(8):1953–60. doi: 10.1093/ckj/sfaa251 PMC8323141 34345419

[17] Rigante D Castellazzi L Bosco A Esposito S. Is There a Crossroad Between Infections, Genetics, and Henoch-Schonlein Purpura? Autoimmun Rev (2013) 12(10):1016–21. doi: 10.1016/j.autrev.2013.04.003 23684700

[18] Weiss PF Klink AJ Luan X Feudtner C. Temporal Association of Streptococcus, Staphylococcus, and Parainfluenza Pediatric Hospitalizations and Hospitalized Cases of Henoch-Schonlein Purpura. J Rheumatol (2010) 37(12):2587–94. doi: 10.3899/jrheum.100364 20843903

[19] Hwang HH Lim IS Choi B-S Yi DY. Analysis of Seasonal Tendencies in Pediatric Henoch–Schönlein Purpura and Comparison With Outbreak of Infectious Diseases. Medicine (2018) 97(36):e12217. doi: 10.1097/MD.0000000000012217 30200139 PMC6133644

[20] Sapina M Frkovic M Sestan M Srsen S Ovuka A Batnozic Varga M. Geospatial Clustering of Childhood IgA Vasculitis and IgA Vasculitis-Associated Nephritis. Ann Rheum Dis (2020) 80:610–6. doi: 10.1136/annrheumdis-2020-218649 33208346

[21] Kiryluk K Li Y Scolari F Sanna-Cherchi S Choi M Verbitsky M. Discovery of New Risk Loci for IgA Nephropathy Implicates Genes Involved in Immunity Against Intestinal Pathogens. Nat Genet (2014) 46(11):1187–96. doi: 10.1038/ng.3118 PMC4213311 25305756

[22] Oni L Sampath S. Childhood IgA Vasculitis (Henoch Schonlein Purpura)-Advances and Knowledge Gaps. Front Pediatr (2019) 7:257. doi: 10.3389/fped.2019.00257 31316952 PMC6610473

[23] Yang YH Hung CF Hsu CR Wang LC Chuang YH Lin YT. A Nationwide Survey on Epidemiological Characteristics of Childhood Henoch-Schonlein Purpura in Taiwan. Rheumatology (2005) 44(5):618–22. doi: 10.1093/rheumatology/keh544 15671050

[24] Xiong LJ Tong Y Wang ZL Mao M. Is Helicobacter Pylori Infection Associated With Henoch-Schonlein Purpura in Chinese Children? A Meta-Analysis. World J Pediatr (2012) 8(4):301–8. doi: 10.1007/s12519-012-0373-1 23151856

[25] Ulas T Tursun I Dal MS Eren MA Buyukhatipoglu H. Rapid Improvement of Henoch-Schonlein Purpura Associated With the Treatment of Helicobacter Pylori Infection. J Res Med Sci (2012) 17(11):1086–8. PMC3702094 23833587

[26] Hoskins B Keeven N Dang M Keller E Nagpal R. A Child With COVID-19 and Immunoglobulin A Vasculitis. Pediatr Ann (2021) 50(1):e44–e8. doi: 10.3928/19382359-20201211-01 33450039

[27] Allez M Denis B Bouaziz JD Battistella M Zagdanski AM Bayart J. COVID-19-Related IgA Vasculitis. Arthritis Rheumatol (2020) 72(11):1952–3. doi: 10.1002/art.41428 PMC7361577 32633104

[28] AlGhoozi DA AlKhayyat HM. A Child With Henoch-Schonlein Purpura Secondary to a COVID-19 Infection. BMJ Case Rep (2021) 14(1):102707. doi: 10.1136/bcr-2020-239910 PMC10577763 33408113

[29] Rasmussen C Tisseyre M Garon-Czmil J Atzenhoffer M Guillevin L Salem JE. Drug-Induced IgA Vasculitis in Children and Adults: Revisiting Drug Causality Using a Dual Pharmacovigilance-Based Approach. Autoimmun Rev (2021) 20(1):102707. doi: 10.1016/j.autrev.2020.102707 33197572

[30] Yang M Li FG Xie XS Wang SQ Fan JM. CagA, a Major Virulence Factor of Helicobacter Pylori, Promotes the Production and Underglycosylation of IgA1 in DAKIKI Cells. Biochem Biophys Res Commun (2014) 444(2):276–81. doi: 10.1016/j.bbrc.2014.01.050 24462875

[31] Yamaguchi H Goto S Takahashi N Tsuchida M Watanabe H Yamamoto S. Aberrant Mucosal Immunoreaction to Tonsillar Microbiota in Immunoglobulin A Nephropathy. Nephrol Dial Transplant (2021) 36(1):75–86. doi: 10.1093/ndt/gfaa223 33099625 PMC7771982

[32] Chang S Li XK. The Role of Immune Modulation in Pathogenesis of IgA Nephropathy. Front Med (Lausanne) (2020) 7:92. doi: 10.3389/fmed.2020.00092 32266276 PMC7105732

[33] Papista C Lechner S Ben Mkaddem S LeStang MB Abbad L Bex-Coudrat J. Gluten Exacerbates IgA Nephropathy in Humanized Mice Through Gliadin-CD89 Interaction. Kidney Int (2015) 88(2):276–85. doi: 10.1038/ki.2015.94 25807036

[34] Davin JC Forget P Mahieu PR. Increased Intestinal Permeability to (51 Cr) EDTA Is Correlated With IgA Immune Complex-Plasma Levels in Children With IgA-Associated Nephropathies. Acta Paediatr Scand (1988) 77(1):118–24. doi: 10.1111/j.1651-2227.1988.tb10609.x 3130743

[35] Noval Rivas M Wakita D Franklin MK Carvalho TT Abolhesn A Gomez AC. Intestinal Permeability and IgA Provoke Immune Vasculitis Linked to Cardiovascular Inflammation. Immunity (2019) 51(3):508–21 e6. doi: 10.1016/j.immuni.2019.05.021 31471109 PMC6751009

[36] Lopez-Mejias R Castaneda S Genre F Remuzgo-Martinez S Carmona FD Llorca J. Genetics of Immunoglobulin-A Vasculitis (Henoch-Schonlein Purpura): An Updated Review. Autoimmun Rev (2018) 17(3):301–15. doi: 10.1016/j.autrev.2017.11.024 29353097

[37] Amoli MM Thomson W Hajeer AH Calvino MC Garcia-Porrua C Ollier WER. HLA-B35 Association With Nephritis in Henoch-Schonlein Purpura. J Rheumatol (2002) 29(5):948–9. 12022355

[38] Amoli MM Thomson W Hajeer AH Calvno MC Garcia-Porrua C Ollier WER. HLA-DRB1*01 Association With Henoch-Schonlein Purpura in Patients From Northwest Spain. J Rheumatol (2001) 28(6):1266–70. 11409118

[39] Lopez-Mejias R Genre F Perez BS Castaneda S Ortego-Centeno N Llorca J. HLA-DRB1 Association With Henoch-Schonlein Purpura. Arthritis Rheumatol (2015) 67(3):823–7. doi: 10.1002/art.38979 25470797

[40] Amoli MM Thomson W Hajeer AH Calvino MC Garcia-Porrua C Ollier WER. Interleukin 8 Gene Polymorphism Is Associated With Increased Risk of Nephritis in Cutaneous Vasculitis. J Rheumatol (2002) 29(11):2367–70. 12415593

[41] Amoli MM Thomson W Hajeer AH Calvino MC Garcia-Porrua C Ollier WER. Interleukin 1 Receptor Antagonist Gene Polymorphism is Associated With Severe Renal Involvement and Renal Sequelae in Henoch-Schonlein Purpura. J Rheumatol (2002) 29(7):1404–7. 12136897

[42] Amoli MM Calvino MC Garcia-Porrua C Llorca J Ollier WER Gonzalez-Gay MA. Interleukin 1 Beta Gene Polymorphism Association With Severe Renal Manifestations and Renal Sequelae in Henoch-Schonlein Purpura. J Rheumatol (2004) 31(2):295–8. 14760799

[43] An J Lu Q Zhao H Cao Y Yan B Ma Z. A Study on the Association Between C1GALT1 Polymorphisms and the Risk of Henoch-Schonlein Purpura in a Chinese Population. Rheumatol Int (2013) 33(10):2539–42. doi: 10.1007/s00296-013-2761-9 23624553

[44] He X Zhao P Kang S Ding Y Luan J Liu Z. C1GALT1 Polymorphisms Are Associated With Henoch-Schonlein Purpura Nephritis. Pediatr Nephrol (2012) 27(9):1505–9. doi: 10.1007/s00467-012-2178-9 22544166

[45] Gershoni-Baruch R Broza Y Brik R. Prevalence and Significance of Mutations in the Familial Mediterranean Fever Gene in Henoch-Schonlein Purpura. J Pediatr (2003) 143(5):658–61. doi: 10.1067/S0022-3476(03)00502-X 14615741

[46] Abbara S Grateau G Ducharme-Benard S Saadoun D Georgin-Lavialle S. Association of Vasculitis and Familial Mediterranean Fever. Front Immunol (2019) 10:763. doi: 10.3389/fimmu.2019.00763 31031761 PMC6473328

[47] Altug U Ensari C Sayin DB Ensari A. MEFV Gene Mutations in Henoch-Schonlein Purpura. Int J Rheum Dis (2013) 16(3):347–51. doi: 10.1111/1756-185X.12072 23981758

[48] Bayram C Demircin G Erdogan O Bulbul M Caltik A Akyuz SG. Prevalence of MEFV Gene Mutations and Their Clinical Correlations in Turkish Children With Henoch-Schonlein Purpura. Acta Paediatr (2011) 100(5):745–9. doi: 10.1111/j.1651-2227.2011.02143.x 21231959

[49] Cakici EK Kurt Sukur ED Ozlu SG Yazilitas F Ozdel S Gur G. MEFV Gene Mutations in Children With Henoch-Schonlein Purpura and Their Correlations-do Mutations Matter? Clin Rheumatol (2019) 38(7):1947–52. doi: 10.1007/s10067-019-04489-2 30826945

[50] Can E Kilinc Yaprak Z Hamilcikan S Erol M Bostan Gayret YOYO. MEFV Gene Mutations and Clinical Course in Pediatric Patients With Henoch-Schonlein Purpura. Arch Argent Pediatr (2018) 116(3):e385–e91. doi: 10.5546/aap.2018.eng.e385 29756710

[51] Dogan CS Akman S Koyun M Bilgen T Comak E Gokceoglu AU. Prevalence and Significance of the MEFV Gene Mutations in Childhood Henoch-Schonlein Purpura Without FMF Symptoms. Rheumatol Int (2013) 33(2):377–80. doi: 10.1007/s00296-012-2400-x 22451026

[52] Nikibakhsh AA Houshmand M Bagheri M Zadeh HM Rad IA. MEFV Gene Mutations (M694V, V726A, M680I, and A744S) in Iranian Children With Henoch-Schonlein Purpura. Pneumologia (2012) 61(2):84–7. 22783597

[53] Salah S Rizk S Lotfy HM El Houchi S Marzouk H Farag Y. MEFV Gene Mutations in Egyptian Children With Henoch-Schonlein Purpura. Pediatr Rheumatol Online J (2014) 12:41. doi: 10.1186/1546-0096-12-41 25232290 PMC4165914

[54] Ekinci RMK Balci S Bisgin A Atmis B Dogruel D Altintas DU. MEFV Gene Variants in Children With Henoch-Schonlein Purpura and Association With Clinical Manifestations: A Single-Center Mediterranean Experience. Postgrad Med (2019) 131(1):68–72. doi: 10.1080/00325481.2019.1552479 30513227

[55] Balci-Peynircioglu B Kaya-Akca U Arici ZS Avci E Akkaya-Ulum ZY Karadag O. Comorbidities in Familial Mediterranean Fever: Analysis of 2000 Genetically Confirmed Patients. Rheumatol (Oxford) (2020) 59(6):1372–80. doi: 10.1093/rheumatology/kez410 31598713

[56] Davin JC Ten Berge IJ Weening JJ. What Is the Difference Between IgA Nephropathy and Henoch-Schonlein Purpura Nephritis? Kidney Int (2001) 59(3):823–34. doi: 10.1046/j.1523-1755.2001.059003823.x 11231337

[57] Saulsbury FT. Clinical Update: Henoch-Schonlein Purpura. Lancet (2007) 369(9566):976–8. doi: 10.1016/s0140-6736(07)60474-7 17382810

[58] Jennette JC. IgA Nephropathy and IgA Vasculitis (Henoch-Schönlein Purpura). In: Fogo AB Cohen AH Colvin RB Jennette JC Alpers CE, editors. Fundamentals of Renal Pathology. Berlin, Heidelberg: Springer Berlin Heidelberg (2014). p. 69–78.

[59] Pillebout E. IgA Vasculitis and IgA Nephropathy: Same Disease? J Clin Med (2021) 10(11):2310. doi: 10.3390/jcm10112310 34070665 PMC8197792

[60] Meadow SR Scott DG. Berger Disease - Henoch-Schonlein Syndrome Without the Rash. J Pediatr (1985) 106(1):27–32. doi: 10.1016/S0022-3476(85)80459-5 2981307

[61] Novak J Rizk D Takahashi K Zhang XW Bian Q Ueda H. New Insights Into the Pathogenesis of IgA Nephropathy. Kidney Dis (2015) 1(1):8–18. doi: 10.1159/000382134 PMC4640461 26568951

[62] Neufeld M Molyneux K Pappelbaum KI Mayer-Hain S von Hodenberg C Ehrchen J. Galactose-Deficient IgA1 in Skin and Serum From Patients With Skin-Limited and Systemic IgA Vasculitis. J Am Acad Dermatol (2019) 81(5):1078–85. doi: 10.1016/j.jaad.2019.03.029 30902725

[63] Tang M Zhang X Li X Lei L Zhang H Ling C. Serum Levels of Galactose-Deficient IgA1 in Chinese Children With IgA Nephropathy, IgA Vasculitis With Nephritis, and IgA Vasculitis. Clin Exp Nephrol (2021) 25(1):37–43. doi: 10.1007/s10157-020-01968-8 32935202

[64] Suzuki H Kiryluk K Novak J Moldoveanu Z Herr AB Renfrow MB. The Pathophysiology of IgA Nephropathy. J Am Soc Nephrol (2011) 22(10):1795–803. doi: 10.1681/asn.2011050464 PMC3892742 21949093

[65] Suzuki H Yasutake J Makita Y Tanbo Y Yamasaki K Sofue T. IgA Nephropathy and IgA Vasculitis With Nephritis Have a Shared Feature Involving Galactose-Deficient IgA1-Oriented Pathogenesis. Kidney Int (2018) 93(3):700–5. doi: 10.1016/j.kint.2017.10.019 29329643

[66] Allen AC Willis FR Beattie TJ Feehally J. Abnormal IgA Glycosylation in Henoch-Schonlein Purpura Restricted to Patients With Clinical Nephritis. Nephrol Dial Transplant (1998) 13(4):930–4. doi: 10.1093/ndt/13.4.930 9568852

[67] Saulsbury FT. Alterations in the O-Linked Glycosylation of IgA1 in Children With Henoch-Schonlein Purpura. J Rheumatol (1997) 24(11):2246–9. 9375892

[68] Jennette JC Stone JR. Chapter 11 - Diseases of Medium-Sized and Small Vessels. In: Willis MS Homeister JW Stone JR, editors. Cellular and Molecular Pathobiology of Cardiovascular Disease. San Diego: Academic Press (2014). p. 197–219.

[69] Mak TW Saunders ME. 20 - Mucosal and Cutaneous Immunity. In: Mak TW Saunders ME, editors. The Immune Response. Burlington: Academic Press (2006). p. 583–609.

[70] Yu H-H Yang Y-H Chiang B-L. Chapter 67 - IgA Nephropathies. In: Shoenfeld Y Meroni PL Gershwin ME, editors. Autoantibodies (Third Edition). San Diego: Elsevier (2014). p. 567–72.

[71] Lai KN Tang SC Schena FP Novak J Tomino Y Fogo AB. IgA Nephropathy. Nat Rev Dis Primers (2016) 2:16001. doi: 10.1038/nrdp.2016.1 27189177

[72] Ohyama Y Yamaguchi H Nakajima K Mizuno T Fukamachi Y Yokoi Y. Analysis of O-Glycoforms of the IgA1 Hinge Region by Sequential Deglycosylation. Sci Rep (2020) 10(1):671. doi: 10.1038/s41598-020-57510-z 31959827 PMC6971281

[73] Reily C Stewart TJ Renfrow MB Novak J. Glycosylation in Health and Disease. Nat Rev Nephrol (2019) 15(6):346–66. doi: 10.1038/s41581-019-0129-4 PMC6590709 30858582

[74] Suzuki H Raska M Yamada K Moldoveanu Z Julian BA Wyatt RJ. Cytokines Alter IgA1 O-Glycosylation by Dysregulating C1GalT1 and ST6GalNAc-II Enzymes. J Biol Chem (2014) 289(8):5330–9. doi: 10.1074/jbc.M113.512277 PMC3931088 24398680

[75] Gale DP Molyneux K Wimbury D Higgins P Levine AP Caplin B. Galactosylation of IgA1 Is Associated With Common Variation in C1GALT1. J Am Soc Nephrol (2017) 28(7):2158–66. doi: 10.1681/ASN.2016091043 PMC5491291 28209808

[76] Gharavi AG Kiryluk K Choi M Li Y Hou P Xie J. Genome-Wide Association Study Identifies Susceptibility Loci for IgA Nephropathy. Nat Genet (2011) 43(4):321–7. doi: 10.1038/ng.787 PMC3412515 21399633

[77] Gharavi AG Moldoveanu Z Wyatt RJ Barker CV Woodford SY Lifton RP. Aberrant IgA1 Glycosylation Is Inherited in Familial and Sporadic IgA Nephropathy. J Am Soc Nephrol (2008) 19(5):1008–14. doi: 10.1681/ASN.2007091052 PMC2386728 18272841

[78] Carney EF. Glomerular Disease: Role of Tonsillar B Cells in IgAN. Nat Rev Nephrol (2017) 13(2):63. doi: 10.1038/nrneph.2016.185 27990017

[79] Muto M Manfroi B Suzuki H Joh K Nagai M Wakai S. Toll-Like Receptor 9 Stimulation Induces Aberrant Expression of a Proliferation-Inducing Ligand by Tonsillar Germinal Center B Cells in IgA Nephropathy. J Am Soc Nephrol (2017) 28(4):1227–38. doi: 10.1681/ASN.2016050496 PMC5373453 27920152

[80] Zheng N Xie K Ye H Dong Y Wang B Luo N. TLR7 in B Cells Promotes Renal Inflammation and Gd-IgA1 Synthesis in IgA Nephropathy. JCI Insight (2020) 5(14):e136965. doi: 10.1172/jci.insight.136965 PMC7453916 32699192

[81] Ciferska H Honsova E Lodererova A Hruskova Z Neprasova M Vachek J. Does the Renal Expression of Toll-Like Receptors Play a Role in Patients With IgA Nephropathy? J Nephrol (2020) 33(2):307–16. doi: 10.1007/s40620-019-00640-z PMC7170228 31489594

[82] Donadio ME Loiacono E Peruzzi L Amore A Camilla R Chiale F. Toll-Like Receptors, Immunoproteasome and Regulatory T Cells in Children With Henoch-Schonlein Purpura and Primary IgA Nephropathy. Pediatr Nephrol (2014) 29(9):1545–51. doi: 10.1007/s00467-014-2807-6 24687448

[83] Heineke MH Ballering AV Jamin A Ben Mkaddem S Monteiro RC Van Egmond M. New Insights in the Pathogenesis of Immunoglobulin A Vasculitis (Henoch-Schonlein Purpura). Autoimmun Rev (2017) 16(12):1246–53. doi: 10.1016/j.autrev.2017.10.009 29037908

[84] Suzuki H Fan R Zhang Z Brown R Hall S Julian BA. Aberrantly Glycosylated IgA1 in IgA Nephropathy Patients Is Recognized by IgG Antibodies With Restricted Heterogeneity. J Clin Invest (2009) 119(6):1668–77. doi: 10.1172/JCI38468 PMC2689118 19478457

[85] Suzuki H Moldoveanu Z Julian BA Wyatt RJ Novak J. Autoantibodies Specific for Galactose-Deficient IgA1 in IgA Vasculitis With Nephritis. Kidney Int Rep (2019) 4(12):1717–24. doi: 10.1016/j.ekir.2019.08.015 PMC6895670 31844808

[86] Zhang X Xie X Shi S Liu L Lv J Zhang H. Plasma Galactose-Deficient Immunoglobulin A1 and Loss of Kidney Function in Patients With Immunoglobulin A Vasculitis Nephritis. Nephrol Dial Transplant (2020) 35(12):2117–23. doi: 10.1093/ndt/gfz151 31377786

[87] Lau KK Suzuki H Novak J Wyatt RJ. Pathogenesis of Henoch-Schonlein Purpura Nephritis. Pediatr Nephrol (2010) 25(1):19–26. doi: 10.1007/s00467-009-1230-x 19526254 PMC2778786

[88] Fellstrom BC Barratt J Cook H Coppo R Feehally J de Fijter JW. Targeted-Release Budesonide *Versus* Placebo in Patients With IgA Nephropathy (NEFIGAN): A Double-Blind, Randomised, Placebo-Controlled Phase 2b Trial. Lancet (2017) 389(10084):2117–27. doi: 10.1016/S0140-6736(17)30550-0 28363480

[89] Venettacci O Larkins N Willis F. Childhood IgA Nephropathy Successfully Treated With Targeted-Release Budesonide: A Case Report. J Paediatr Child Health (2018) 54(12):1403. doi: 10.1111/jpc.14259 30506786

[90] Fellstrom B Barratt J Cook H Coppo R Feehally J defijter J. Proteinuria Reduction in Iga Nephropathy by Nefecon, a Targeted Release Formulation of Budesonide - Results From the Nefigan Trial. Nephrol Dialysis Transplant (2017) 32:82–+. doi: 10.1093/ndt/gfx129.TO013

[91] Yanagihara T Brown R Hall S Moldoveanu Z Goepfert A Tomana M. *In Vitro*-Generated Immune Complexes Containing Galactose-Deficient IgA1 Stimulate Proliferation of Mesangial Cells. Results Immunol (2012) 2:166–72. doi: 10.1016/j.rinim.2012.08.002 PMC3775378 24052934

[92] Kiryluk K Moldoveanu Z Sanders JT Eison TM Suzuki H Julian BA. Aberrant Glycosylation of IgA1 Is Inherited in Both Pediatric IgA Nephropathy and Henoch-Schonlein Purpura Nephritis. Kidney Int (2011) 80(1):79–87. doi: 10.1038/ki.2011.16 21326171 PMC3641561

[93] Davin JC Coppo R. Henoch-Schonlein Purpura Nephritis in Children. Nat Rev Nephrol (2014) 10(10):563–73. doi: 10.1038/nrneph.2014.126 25072122

[94] Levinsky RJ Barratt TM. IgA Immune Complexes in Henoch-Schönlein Purpura. Lancet (London England) (1979) 2(8152):1100–3. doi: 10.1016/S0140-6736(79)92505-4 91839

[95] Levinsky RJ Barratt TM. IgA Immune Complexes in Henoch- Schölein Purpura. Lancet (1979) 314(8152):1100–3. doi: 10.1016/S0140-6736(79)92505-4 91839

[96] Berthelot L Jamin A Viglietti D Chemouny JM Ayari H Pierre M. Value of Biomarkers for Predicting Immunoglobulin A Vasculitis Nephritis Outcome in an Adult Prospective Cohort. Nephrol Dialysis Transplant (2018) 33(9):1579–90. doi: 10.1093/ndt/gfx300 29126311

[97] Pillebout E Jamin A Ayari H Housset P Pierre M Sauvaget V. Biomarkers of IgA Vasculitis Nephritis in Children. PloS One (2017) 12(11):e0188718. doi: 10.1371/journal.pone.0188718 29190714 PMC5708800

[98] Wines BD Sardjono CT Trist HH Lay CS Hogarth PM. The Interaction of Fc Alpha RI With IgA and Its Implications for Ligand Binding by Immunoreceptors of the Leukocyte Receptor Cluster. J Immunol (2001) 166(3):1781–9. doi: 10.4049/jimmunol.166.3.1781 11160224

[99] Monteiro RC. Role of IgA and IgA Fc Receptors in Inflammation. J Clin Immunol (2010) 30(1):1–9. doi: 10.1007/s10875-009-9338-0 19834792

[100] Breedveld A van Egmond M. IgA and Fc Alpha RI: Pathological Roles and Therapeutic Opportunities. Front Immunol (2019) 10:553. doi: 10.3389/fimmu.2019.00553 30984170 PMC6448004

[101] Perse M Veceric-Haler Z. The Role of IgA in the Pathogenesis of IgA Nephropathy. Int J Mol Sci (2019) 20(24):6199. doi: 10.3390/ijms20246199 PMC6940854 31818032

[102] Moura IC Arcos-Fajardo M Sadaka C Leroy V Benhamou M Novak J. Glycosylation and Size of IgA1 Are Essential for Interaction With Mesangial Transferrin Receptor in IgA Nephropathy. J Am Soc Nephrol (2004) 15(3):622–34. doi: 10.1097/01.asn.0000115401.07980.0c 14978164

[103] Tomana M Novak J Julian BA Matousovic K Konecny K Mestecky J. Circulating Immune Complexes in IgA Nephropathy Consist of IgA1 With Galactose-Deficient Hinge Region and Antiglycan Antibodies. J Clin Invest (1999) 104(1):73–81. doi: 10.1172/JCI5535 10393701 PMC408399

[104] Xu L Li B Huang M Xie K Li D Li Y. Critical Role of Kupffer Cell CD89 Expression in Experimental IgA Nephropathy. PloS One (2016) 11(7):e0159426. doi: 10.1371/journal.pone.0159426 27437939 PMC4954728

[105] Rifai A Fadden K Morrison SL Chintalacharuvu KR. The N-Glycans Determine the Differential Blood Clearance and Hepatic Uptake of Human Immunoglobulin (Ig)A1 and IgA2 Isotypes. J Exp Med (2000) 191(12):2171–82. doi: 10.1084/jem.191.12.2171 PMC2193211 10859341

[106] Berthelot L Papista C Maciel TT Biarnes-Pelicot M Tissandie E Wang PH. Transglutaminase Is Essential for IgA Nephropathy Development Acting Through IgA Receptors. J Exp Med (2012) 209(4):793–806. doi: 10.1084/jem.20112005 22451718 PMC3328362

[107] Zhang Q Yan L Chen M Gui M Lu L Deng F. IgA1 Isolated From Henoch-Schonlein Purpura Children Promotes Proliferation of Human Mesangial Cells *In Vitro* . Cell Biol Int (2019) 43(7):760–9. doi: 10.1002/cbin.11142 30958627

[108] Molyneux K Wimbury D Pawluczyk I Muto M Bhachu J Mertens PR. Beta1,4-Galactosyltransferase 1 Is a Novel Receptor for IgA in Human Mesangial Cells. Kidney Int (2017) 92(6):1458–68. doi: 10.1016/j.kint.2017.05.002 28750925

[109] Knoppova B Reily C Maillard N Rizk DV Moldoveanu Z Mestecky J. The Origin and Activities of IgA1-Containing Immune Complexes in IgA Nephropathy. Front Immunol (2016) 7:117. doi: 10.3389/fimmu.2016.00117 27148252 PMC4828451

[110] Robert T Berthelot L Cambier A Rondeau E Monteiro RC. Molecular Insights Into the Pathogenesis of IgA Nephropathy. Trends Mol Med (2015) 21(12):762–75. doi: 10.1016/j.molmed.2015.10.003 26614735

[111] Yasutake J Suzuki Y Suzuki H Hiura N Yanagawa H Makita Y. Novel Lectin-Independent Approach to Detect Galactose-Deficient IgA1 in IgA Nephropathy. Nephrology dialysis Transplant Off Publ Eur Dialysis Transplant Assoc - Eur Renal Assoc (2015) 30(8):1315–21. doi: 10.1093/ndt/gfv221 PMC4513896 26109484

[112] Cassol CA Bott C Nadasdy GM Alberton V Malvar A Nagaraja HN. Immunostaining for Galactose-Deficient Immunoglobulin A Is Not Specific for Primary Immunoglobulin A Nephropathy. Nephrology dialysis Transplant Off Publ Eur Dialysis Transplant Assoc - Eur Renal Assoc (2020) 35(12):2123–9. doi: 10.1093/ndt/gfz152 31369128

[113] Zhao L Peng L Yang DY Chen S Lan ZX Zhu XJ. Immunostaining of Galactose-Deficient IgA1 by KM55 Is Not Specific for Immunoglobulin A Nephropathy. Clin Immunol (2020) 217:108483. doi: 10.1016/j.clim.2020.108483 32479989

[114] Yang Y-H Tsai IJ Chang C-J Chuang Y-H Hsu H-Y Chiang B-L. The Interaction Between Circulating Complement Proteins and Cutaneous Microvascular Endothelial Cells in the Development of Childhood Henoch-Schönlein Purpura. PloS One (2015) 10(3):e0120411. doi: 10.1371/journal.pone.0120411 25760949 PMC4356510

[115] Chua JS Zandbergen M Wolterbeek R Baelde HJ van Es LA de Fijter JW. Complement-Mediated Microangiopathy in IgA Nephropathy and IgA Vasculitis With Nephritis. Modern Pathol an Off J United States Can Acad Pathol Inc (2019) 32(8):1147–57. doi: 10.1038/s41379-019-0259-z 30936425

[116] Roos A Bouwman LH van Gijlswijk-Janssen DJ Faber-Krol MC Stahl GL Daha MR. Human IgA Activates the Complement System *via* the Mannan-Binding Lectin Pathway. J Immunol (2001) 167(5):2861–8. doi: 10.4049/jimmunol.167.5.2861 11509633

[117] Hisano S Matsushita M Fujita T Iwasaki H. Activation of the Lectin Complement Pathway in Henoch-Schonlein Purpura Nephritis. Am J Kidney Dis (2005) 45(2):295–302. doi: 10.1053/j.ajkd.2004.10.020 15685507

[118] Endo M Ohi H Ohsawa I Fujita T Matsushita M. Complement Activation Through the Lectin Pathway in Patients With Henoch-Schonlein Purpura Nephritis. Am J Kidney Dis (2000) 35(3):401–7. doi: 10.1016/s0272-6386(00)70192-2 10692265

[119] Selvaskandan H Kay Cheung C Dormer J Wimbury D Martinez M Xu G. Inhibition of the Lectin Pathway of the Complement System as a Novel Approach in the Management of IgA Vasculitis-Associated Nephritis. Nephron (2020) 144(9):453–8. doi: 10.1159/000508841 32721954

[120] van der Steen L Tuk CW Bakema JE Kooij G Reijerkerk A Vidarsson G. Immunoglobulin A: Fc(alpha)RI Interactions Induce Neutrophil Migration Through Release of Leukotriene B4. Gastroenterology (2009) 137(6):2018–29 e1-3. doi: 10.1053/j.gastro.2009.06.047 19555692

[121] Aleyd E van Hout MW Ganzevles SH Hoeben KA Everts V Bakema JE. IgA Enhances NETosis and Release of Neutrophil Extracellular Traps by Polymorphonuclear Cells *via* Fcalpha Receptor I. J Immunol (2014) 192(5):2374–83. doi: 10.4049/jimmunol.1300261 24493821

[122] Papayannopoulos V. Neutrophil Extracellular Traps in Immunity and Disease. Nat Rev Immunol (2018) 18(2):134–47. doi: 10.1038/nri.2017.105 28990587

[123] Behnen M Leschczyk C Moller S Batel T Klinger M Solbach W. Immobilized Immune Complexes Induce Neutrophil Extracellular Trap Release by Human Neutrophil Granulocytes *via* FcgammaRIIIB and Mac-1. J Immunol (2014) 193(4):1954–65. doi: 10.4049/jimmunol.1400478 25024378

[124] Aleyd E Al M Tuk CW van der Laken CJ van Egmond M. IgA Complexes in Plasma and Synovial Fluid of Patients With Rheumatoid Arthritis Induce Neutrophil Extracellular Traps *via* FcalphaRI. J Immunol (2016) 197(12):4552–9. doi: 10.4049/jimmunol.1502353 27913645

[125] Bergqvist C Safi R El Hasbani G Abbas O Kibbi A Nassar D. Neutrophil Extracellular Traps Are Present in Immune-Complex-Mediated Cutaneous Small Vessel Vasculitis and Correlate With the Production of Reactive Oxygen Species and the Severity of Vessel Damage. Acta Derm Venereol (2020) 100(17):adv00281. doi: 10.2340/00015555-3363 31663600 PMC9274929

[126] Imai T Nishiyama K Ueki K Tanaka T Kaku Y Hara T. Involvement of Activated Cytotoxic T Lymphocytes and Natural Killer Cells in Henoch-Schonlein Purpura Nephritis. Clin Transl Immunol (2020) 9(11):e1212. doi: 10.1002/cti2.1212 PMC7684975 33282293

[127] Audemard-Verger A Pillebout E Jamin A Berthelot L Aufray C Martin B. Recruitment of CXCR3+ T Cells Into Injured Tissues in Adult IgA Vasculitis Patients Correlates With Disease Activity. J Autoimmun (2019) 99:73–80. doi: 10.1016/j.jaut.2019.01.012 30745186

[128] Jen HY Chuang YH Lin SC Chiang BL Yang YH. Increased Serum Interleukin-17 and Peripheral Th17 Cells in Children With Acute Henoch-Schonlein Purpura. Pediatr Allergy Immunol (2011) 22(8):862–8. doi: 10.1111/j.1399-3038.2011.01198.x 21929599

[129] Jaszczura M Mizgała-Izworska E Świętochowska E Machura E. Serum Levels of Selected Cytokines [Interleukin (IL)-17a, IL-18, IL-23] and Chemokines (RANTES, IP10) in the Acute Phase of Immunoglobulin A Vasculitis in Children. Rheumatol Int (2019) 39(11):1945–53. doi: 10.1007/s00296-019-04415-4 PMC7575498 31468124

[130] Perkovic D Simac P Katic J. IgA Vasculitis During Secukinumab Therapy. Clin Rheumatol (2020) 40:2071–73. doi: 10.1007/s10067-020-05364-1 32860543

[131] Fukuda M Nobeyama Y Asahina A. Antigenic Competition: IgA Vasculitis Distributing Away From Psoriatic Plaque. J Dermatol (2021) 48(3):e130–e1. doi: 10.1111/1346-8138.15742 33368676

[132] Zeng H Zhang R Jin B Chen L. Type 1 Regulatory T Cells: A New Mechanism of Peripheral Immune Tolerance. Cell Mol Immunol (2015) 12(5):566–71. doi: 10.1038/cmi.2015.44 PMC4579656 26051475

[133] Roncarolo MG Bacchetta R Bordignon C Narula S Levings MK. Type 1 T Regulatory Cells. Immunol Rev (2001) 182(1):68–79. doi: 10.1034/j.1600-065X.2001.1820105.x 11722624

[134] Pan L Wang J Liu J Guo L Yang S. Deficiency in the Frequency and Function of Tr1 Cells in IgAV and the Possible Role of IL-27. Rheumatol (Oxford) (2020) 60(7):3432–42. doi: 10.1093/rheumatology/keaa752 PMC8516516 33280050

[135] Wu H Wen Y Yue C Li X Gao R. Serum TNF- Level Is Associated With Disease Severity in Adult Patients With Immunoglobulin A Vasculitis Nephritis. Dis Markers (2020) 2020:5514145. doi: 10.1155/2020/5514145 33299497 PMC7710402

[136] Villatoro-Villar M Crowson CS Warrington KJ Makol A Koster MJ. Immunoglobulin A Vasculitis Associated With Inflammatory Bowel Disease: A Retrospective Cohort Study. Scand J Rheumatol (2021) 50(1):40–7. doi: 10.1080/03009742.2020.1732460 32456601

[137] Saint Marcoux B De Bandt M Cri. Vasculitides Induced by TNFalpha Antagonists: A Study in 39 Patients in France. Joint Bone Spine (2006) 73(6):710–3. doi: 10.1016/j.jbspin.2006.02.010 17127088

[138] Marques I Lagos A Reis J Pinto A Neves B. Reversible Henoch-Schonlein Purpura Complicating Adalimumab Therapy. J Crohns Colitis (2012) 6(7):796–9. doi: 10.1016/j.crohns.2012.02.019 22445079

